# Antidiabetic Indian Plants: A Good Source of Potent Amylase Inhibitors

**DOI:** 10.1093/ecam/nen040

**Published:** 2011-05-02

**Authors:** Menakshi Bhat, Smita S. Zinjarde, Shobha Y. Bhargava, Ameeta Ravi Kumar, Bimba N. Joshi

**Affiliations:** ^1^Institute of Bioinformatics and Biotechnology, University of Pune, Pune 411 007, India; ^2^Department of Zoology, University of Pune, Pune 411 007, India

## Abstract

Diabetes is known as a multifactorial disease. The treatment of diabetes (Type II) is complicated due to the inherent patho-physiological factors related to this disease. One of the complications of diabetes is post-prandial hyperglycemia (PPHG). Glucosidase inhibitors, particularly **α**-amylase inhibitors are a class of compounds that helps in managing PPHG. Six ethno-botanically known plants having antidiabetic property namely, *Azadirachta indica* Adr. Juss.; *Murraya koenigii* (L.) Sprengel; *Ocimum tenuflorum* (L.) (syn: *Sanctum*); *Syzygium cumini* (L.) Skeels (syn: *Eugenia jambolana*); *Linum usitatissimum* (L.) and *Bougainvillea spectabilis* were tested for their ability to inhibit glucosidase activity. The chloroform, methanol and aqueous extracts were prepared sequentially from either leaves or seeds of these plants. It was observed that the chloroform extract of *O. tenuflorum; B. spectabilis; M. koenigii* and *S. cumini* have significant **α**-amylase inhibitory property. Plants extracts were further tested against murine pancreatic, liver and small intestinal crude enzyme preparations for glucosidase inhibitory activity. The three extracts of *O. tenuflorum* and chloroform extract of *M. koenigi* showed good inhibition of murine pancreatic and intestinal glucosidases as compared with acarbose, a known glucosidase inhibitor.

## 1. Introduction

Diabetes is defined as a state in which homeostasis of carbohydrate and lipid metabolism is improperly regulated by the pancreatic hormone, insulin; resulting in an increased blood glucose level. Diabetes is a progressive disease and is one of the major killers in recent times. World Health Organization (WHO) suggests that worldwide the global population is in the midst of a diabetes epidemic with people in Southeast Asia and Western Pacific being mostly at risk. The number of cases for diabetes that is currently at 171 million is predicted to reach 366 million by the year 2030 [1].

Most prevalent form of diabetes is non-insulin dependent diabetes mellitus (NIDDM/type II). The treatment of type II diabetes is complicated by several factors inherent to the disease and elevated post prandial hyperglycemia (PPHG) is one of the risk factors [2]. PPHG is elevated by the action of glucosidases, a class of enzymes that helps in the breakdown of complex carbohydrates into simple sugars such as maltose and glucose. Glucosidase inhibitors such as *α*-amylase inhibitors plays a major role in managing PPHG in diabetic patients. These *α*-amylase inhibitors inhibit the action of *α*-amylase enzyme leading to a reduction in starch hydrolysis which shows beneficial effects on glycemic index control in diabetic patients [3]. The known glucosidase inhibitors which are conventionally used in the management of diabetes are acarbose and miglitol (a deoxy nojirimycin derivative) that competitively and reversibly inhibits *α*-glucosidase enzyme from intestine as well as pancreas, however, both these drugs are known to be associated with gastrointestinal side effects such as abdominal pain, flatulence and diarrhea in the patients [4, 5]. Therefore, it becomes necessary to identify the amylase inhibitors from natural sources having lesser side-effects.

Ayurveda, the traditional Indian herbal medicinal system practiced for over thousands of years have reports of antidiabetic plants with no known side effects. Such plants and their products have been widely prescribed for diabetic treatment all around the world with less known mechanistic basis of their functioning. Hence, these natural products need to be evaluated scientifically and methodically in order to check for their properties [6, 7].

We tested six plants namely *Azadirachta indica*; *Syzygium cumini*; *Ocimum tenuflorum*; *Murraya koenigii*; *Linum usitatissimum* traditionally used in Ayurveda along with *Bougainvillea spectabilis* used as a hypoglycemic plant in West Indies and some parts of Asia. Most of the plants tested in this study are part of dietary component, so there is less possibility of side effects caused by these plants. Of the six plants tested, *A. indica* and *S. cumini* are earlier reported to possess *α*-amylase inhibitory activity [8]. The extracts of all these plants were tested for their ability to inhibit the enzymatic activity of commercially available porcine pancreatic *α*-amylase and glucosidases from pancreas, liver and small intestine of Swiss mice.

## 2. Materials and Methods

### 2.1. Chemicals

DNSA (dinitrosalicylic acid), porcine pancreatic *α*-amylase and chloroform were obtained from SRL Pvt. Ltd (Mumbai, India). Di-potassium hydrogen phosphate (K_2_HPO_4_), potassium dihydrogen phosphate (KH_2_PO_4_), methanol, sodium potassium tartarate, sodium hydroxide (NaOH), were obtained from Merck Chemicals Ltd (Mumbai, India). Sodium chloride (NaCl) was obtained from HiMedia Laboratories (Mumbai, India). Acarbose was obtained from Bayer Pharmaceuticals Pvt. Ltd (Mumbai, India). All the chemicals and reagents procured were of A.R. grade.

### 2.2. Plant Materials

Leaves of *A. indica, M. koenigii*, *O. tenuflorum*, *B. spectabilis* and seeds of *L. usitatissimum* and *S. cumini* were collected in and around Pune city in the months of February, March and June. All the plants were authenticated by Botanical Survey of India (BSI), Pune, India.

### 2.3. Preparation of Plant Extracts

The leaves were washed and air dried at room temperature. The chloroform, methanol and aqueous extracts were prepared sequentially in soxhlet extractor using 30 g of dried plant tissue mixed with 150 ml of the respective solvent (100% v/v) for 24 h [9, 10]. Methanolic and chloroform extracts were evaporated to dryness in rotary evaporator, whereas the aqueous extracts were lyophilized [11]. 25 mg dry weight of each crude extract was further reconstituted in 2.5 ml of distilled water and 1 : 20 dilution of all these extracts were used for further studies.

### 2.4. Preparation of Murine Pancreatic, Liver and Small Intestine Extracts

The 10-week-old Swiss male mice were procured from the animal house of Department of Zoology. The entire procedure was carried out with guidelines of Institutional Animal Ethical Committee. The mouse weighing 27 g was starved for 12 h. Pancreas; liver and small intestine tissues were excised and homogenized with 10 mM ice cold phosphate buffer containing 100 mM NaCl (1 : 10 dilution; w/v) and appropriate amount of protease inhibitors. The homogenate was centrifuged for 10 min at 10 000 r.p.m. and the supernatant was taken as a source of the enzyme [12].

### 2.5. *α*-Amylase Inhibition Assay

The 45.5 *μ*g ml^−1^ of porcine pancreatic *α*-amylase was incubated with 25 mg ml^−1^ plant extracts. One percent starch was used as substrate. The plant extracts without *α*-amylase were used as controls and the test reading were subtracted from the absorbance of these controls. The reducing sugar was estimated using DNSA assay at A_540_ nm and the enzyme units were expressed as micro molar per minute [13]. One unit of enzyme was defined as the amount of enzyme required to liberate 1 *μ*M of maltose under assay conditions. The final inhibition shown by different crude extracts were compared with the standard inhibitor, 1.9 mM acarbose. The 50% inhibition concentration (IC_50_) of plant extracts against porcine pancreatic *α*-amylase was also calculated.

### 2.6. Glucosidase Inhibition Assay with Murine Pancreatic, Liver and Small Intestinal Extracts

Supernatant obtained from pancreas, liver and small intestine tissues were taken as a source of enzyme and were diluted so as to get an absorbance of 0.4 (at 280 nm) which corresponds to 11.52, 3.62, and 46.40 *μ*g of protein respectively. Protein was estimated using Bradford standard curve [14]. The enzyme inhibition assay was carried out as described above. The 50% inhibition concentration (IC_50_) of plant extracts against pancreatic, small intestinal and liver glucosidases was calculated.

### 2.7. Statistical Analysis

The statistical analysis was performed using one way analysis of variance (ANOVA) and two tailed *t*-test (*P* <  .05). Results are expressed as means ± SEM *n* = 3.

## 3. Results

### 3.1. Porcine Pancreatic *α*-Amylase Inhibitory Activity of Plant Extracts

**Figure 1:**
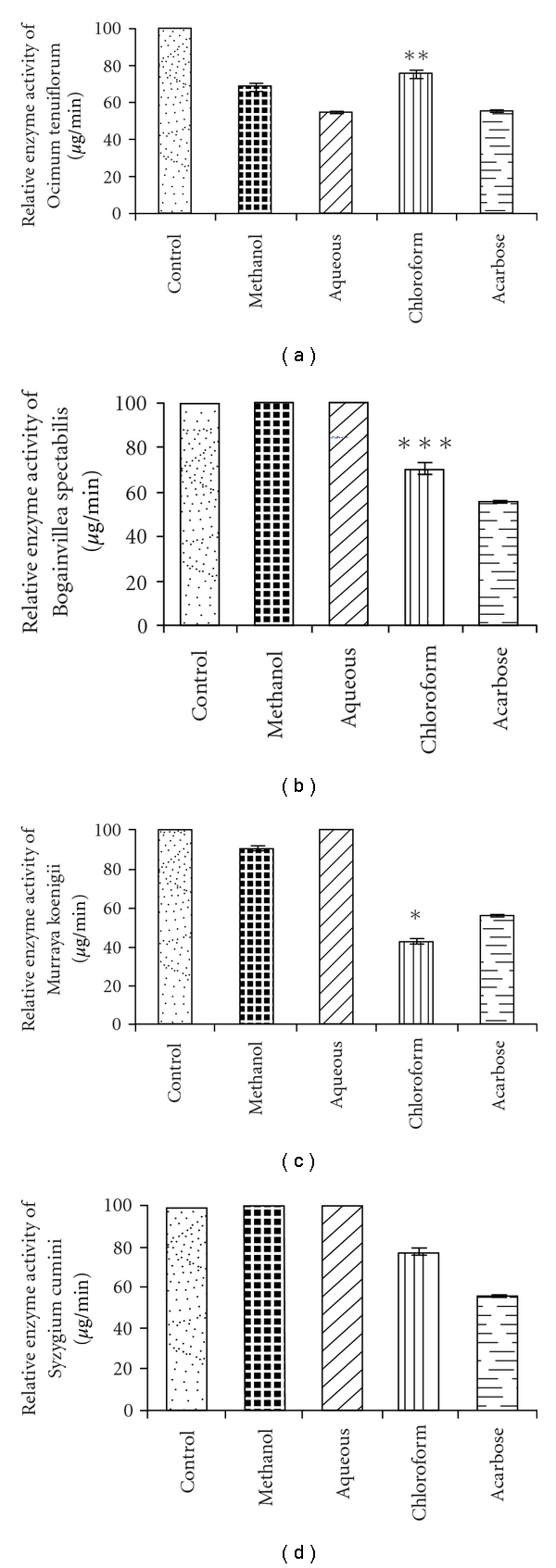
The percent relative enzyme activity after inhibition with (a) *M. koenigii* (b) *B. spectabilis* (c) *O. tenuflorum* (d) *S. cumini*, extracts on porcine pancreatic *α*-amylase. Acarbose is taken as standard *α*-amylase inhibitor. Pure porcine pancreatic *α*-amylase serves as control. The data is indicated as the mean ± SEM; (*n* = 3). Data with different asterisks (*, **, ***) shows significant difference with (*) denoting more significant value (*P* <  .05), two-tailed student *t*-test.

Porcine pancreatic *α*-amylase with 0.21 U min^−1^ was taken as 100% enzymatic activity. Chloroform extract of *M. koenigii, B. spectabilis, O. tenuflorum* and *S. cumini* showed enzyme inhibition of 56.64, 29.43, 24.57 and 22.31% respectively (Figure 1). A significant inhibition was observed with extracts of *O. tenuflorum;* whereas *A. indica* and *L. usitatissimum* showed no inhibition with pure porcine *α*-amylase. The chloroform extract of *M. koenigii* showed significant difference with *P* <  .05 as compared with other plant extracts (Figure 1).

### 3.2. Murine Pancreatic Glucosidases Inhibitory Activity of Plant Extracts

Murine pancreatic tissue weighing 1.2 g with 72.0 *μ*g ml^−1^ of total protein was used as a source of enzyme, of which 11.5 *μ*g of protein was used for inhibition assay. Pancreatic enzyme activity exhibiting 0.26 U min^−1^ was taken as 100% enzymatic activity. Chloroform extracts of *M. koenigii, B. spectabilis* and *S. cumini* showed enzyme inhibitory activity of 83.94, 51.99 and 43.28% and aqueous extracts of *O. tenuflorum*, *L. usitatissimum* and *B. spectabilis* showed enzyme inhibitory activity of 76.27, 61.68 and 55.32%, respectively as compared to acarbose which showed 40.43% enzyme inhibition. The plant extracts of *A. indica* and *L. usitatissimum* showed negligible enzyme inhibition. The chloroform extract of *O. tenuflorum* showed significant difference with *P* <  .05 as compared with other plant extracts (Table 1).

### 3.3. Murine Small Intestinal Glucosidases Inhibitory Activity of Plant Extracts

Murine small intestine tissue weighing 0.6 g with 145 *μ*g ml^−1^ total protein was used as a source of enzyme of which 46.40 *μ*g of protein was used for inhibition assay. Intestinal enzyme activity exhibiting 0.03 U min^−1^ was taken as 100% enzymatic activity. Aqueous and methanolic extracts of *A. indica* showed enzyme inhibition of 62.44 and 41.07% while *M. koenigii* chloroform extract showed enzyme inhibition of 60.99 and 43.72%, respectively, followed by *S. cumini* chloroform extracts with 44.40% enzyme inhibition. However, acarbose failed to show any inhibition with intestinal glucosidase. The chloroform extract of *M. koenigii* showed significant difference with *P* <  .05 as compared with other plant extracts (Table 2).

### 3.4. Murine Liver Glucosidases Inhibitory Activity of Plant Extracts

Murine liver tissue extract weighing 1.8 g with 181 *μ*g ml^−1^ total protein was used as a source of enzyme, of which 3.62 *μ*g of protein was used for inhibition assay. Liver enzyme activity exhibiting 0.06 U min^−1^ was taken as 100% enzymatic activity. The methanolic extract of *S. cumini, A. indica* and *B. spectabilis* showed inhibition of 55.09, 52.11 and 33.09% respectively, whereas the aqueous extracts of *A. indica* and *O. tenuflorum* showed enzyme inhibition of 69.29 and 26.63%, respectively. The chloroform extract of *B. spectabilis* showed significant difference with *P* <  .05 as compared with other plant extracts (Table 3).

## 4. Discussion

There are many reports of herbal extracts being used in Ayurvedic literature as antidiabetic which are directly or indirectly used for the preparation of many modern drugs. However, these plants have not gained much importance as medicines and one of the factors is lack of specific standards being prescribed for herbal medicines and supportive animal/clinical trials [15]. In the present study, we investigate six known plants, namely *A. indica*; *S. cumini*; *O. tenuflorum*; *M. koenigii*; *L. usitatissimum* and *B. spectabilis* having antidiabetic properties for their possible glucosidase inhibitory activity. Plants are known to produce a large variety of glucosidase inhibitors that provides protection against insects and microbial pathogens [16, 17] therefore plant extracts were analyzed for *α*-amylase inhibitory activity.

Pancreatic and intestinal glucosidases are the key enzymes of dietary carbohydrate digestion and inhibitors of these enzymes may be effective in retarding glucose absorption to suppress PPHG. In diabetic state, excessive hepatic glycogenolysis and gluconeogenesis is associated with decreased utilization of glucose by tissues being the fundamental mechanism underlying hyperglycemia [18]. The liver glucosidase inhibitors, inhibits *α*-1,6-glucosidase of glycogen-debranching enzymes in the liver and reduces the glycogenolytic rate which increases the accumulation of glycogen stores in the liver [19, 20]. Inhibition of these enzyme systems decreases the current blood glucose levels in diabetic patient (as a short term effect) and shows a small reduction in hemoglobin _A1c_ level (as a long term effect) [21].

Earlier studies indicated that *M. koenigii* possess hypoglycemic activity, decreased glycogenolysis and gluconeogenesis properties and also finds its application as an adjuvant to dietary therapy and drug treatment for controlling Diabetes Mellitus [22–24]. Our investigation reported *M. koenigii* as a good glucosidases inhibitor showing inhibition against porcine pancreatic *α*-amylase as well as murine pancreatic and intestinal glucosidases. The chloroform extract of *M. koenigii* showed significant inhibition (IC_50_ values of 1.96, 1.06 and 2.68 *μ*g ml^−1^) with porcine pancreatic *α*-amylase (56.40%) as well as murine pancreatic and intestinal glucosidases, respectively, which is significantly higher than acarbose. Interestingly the phenolic content of this extract was very low, so the inhibitory activity may be because of specific inhibitory compounds. Methanolic extract showed inhibition (IC_50_ value of 1.96 *μ*g ml^−1^) only with murine intestinal glucosidase as shown in Table 2.

Similarly *O. tenuflorum* shows inhibition of *α*-amylase known to possess antidiabetic properties [25, 26]. The three extracts of *O. tenuflorum* shows inhibitory activity against porcine *α*-amylase, murine pancreatic and liver glucosidases. Chloroform extract possess more potent inhibitory activity (IC_50_ value of 1.76 *μ*g ml^−1^) with murine pancreatic glucosidases. The inhibitory activity of aqueous extract (IC_50_ values of 1.55 and 9.86 *μ*g ml^−1^) was observed with porcine *α*-amylase and with murine liver glucosidases, respectively, which is significantly more than acarbose.

*Syzygium cumini* is known to possess properties of normoglycemia and lowers blood glucose levels by improving oral glucose tolerance [27, 28]. Our study suggests *S. cumini* as a good inhibitor of glucosidases. Inhibitory activity of chloroform extract (IC_50_ values of 4.28, 3.72 and 5.60 *μ*g ml^−1^) was observed against porcine *α*-amylase, murine pancreatic and murine intestinal glucosidases, respectively. The methanolic extract of *S. cumini* showed inhibition with only murine liver glucosidases (IC_50_ = 2.68 *μ*g ml^−1^).

*Bougainvillea spectabilis* on the other hand is being used for the treatment of diabetes in the islands of West Indies and Asia. It is claimed to exert insulin-like effects and contain D-pinitol (3-O-methylchiroinositol) [29–31]. Our studies indicated *B. spectabilis* chloroform extract has effective inhibition (IC_50_ values of 3.20 and 2.06 *μ*g ml^−1^) on porcine pancreatic *α*-amylase and murine pancreatic glucosidases. The aqueous extract showed inhibition (IC_50_ = 11.16 *μ*g ml^−1^) with murine pancreatic glucosidases and methanolic extract (IC_50_ = 9.26 *μ*g ml^−1^) showed inhibition with murine liver glucosidase. The effects could be possibly because of the high-phenolic contents present in the crude extracts.

*Azadirachta indica* is one of the widely used antidiabetic plants, which possess hypolipidaemic, hypoglycaemic, immunostimulant and hepatoprotective properties [32–34]. Aqueous extracts of neem leaves was reported to reduce blood glucose and this effect could be due to blocking the action of epinephrine on glycogenolysis and peripheral utilization of glucose [35]. It is not a glucosidase inhibitor as evidenced by no significant inhibition with porcine pancreatic *α*-amylase. However, methanolic (IC_50_ values of 2.60 and 1.80 *μ*g ml^−1^) and aqueous extract (IC_50_ values of 3.17 and 6.21 *μ*g ml^−1^) showed inhibition with murine liver and intestinal glucosidases, respectively, which is significantly more than acarbose.

*Linum usitatissimum* is known to possess plasma triglycerides and cholesterol lowering properties. This plant being a dietary component is also known to have hypolipidemic effect [36]. We investigate its role as glucosidase inhibitor. The aqueous (IC_50_ value of 3.21 *μ*g ml^−1^) and methanolic extract (IC_50_ value of 18.62 *μ*g ml^−1^) has shown inhibition with murine pancreatic glucosidases which is significantly more than acarbose.

Diabetes being multifactorial disease the treatment choice differs from patient to patient. Therefore, it is important to find out the biological activity of these herbal extracts. Theoretically, these glucosidase inhibitors from plants act through a variety of mechanism. Presently it is not possible either to pin point the exact mechanism of inhibition of these extracts or to identify the active principle(s) responsible for such effect in this study. Nevertheless, the present study identifies the *M. koenigii* and *O. tenuflorum* as good glucosidase inhibitors of pancreatic and intestinal enzymes. They can be further studied and may be used as dietary supplement for controlling PPHG in type II diabetes.

## 5. Conclusion

*M. koenigii* and *O. tenuflorum* shows significant inhibition with porcine pancreatic, murine pancreatic as well as intestine glucosidase. This may have beneficial effects in managing type II diabetes mellitus and could be used as an indicator for combinational therapy which can be taken up in further studies.

## Figures and Tables

**Table 1:** Percent enzyme inhibition shown by plant extracts with pancreatic glucosidases.

Acarbose (40.43 ± 1.22)	% Glucosidases inhibition activity		
	Methanol	Aqueous	Chloroform
*Murraya koenigii* (L.) Sprengel	ND	ND	83.93 ± 1.55*
*Bougainvillea spectabilis*	ND	55.31 ± 5.09	51.98 ± 6.07
*Ocimum tenuiflorum* (L.)	ND	76.27 ± 3.67**	ND
*Syzygium cumini* (L.) Skeels	ND	ND	43.28 ± 1.48
*Azadirachta indica* Adr. Juss	ND	ND	ND
*Linum usitatissimum* (L.)	26.01 ± 3.18	61.67 ± 2.86**	ND

The table shows percent inhibition of pancreatic glucosidases using plant extracts. The data is indicated as the mean ± SEM; (*n* = 3). Two-tailed *t*-test was applied. Values with different asterisk (*, **) shows significant difference with (*) denoting more significant value, *P* <  .05, two-tailed student *t*-test. ND defines no enzyme inhibition.

**Table 2:** Percent enzyme inhibition shown by plant extracts with small intestinal glucosidases.

Acarbose (ND)	% Glucosidases inhibition activity		
	Methanol	Aqueous	Chloroform
*Murraya koenigii* (L.) Sprengel	43.71 ± 2.42	ND	60.99 ± 3.20**
*Bougainvillea spectabilis*	ND	ND	ND
*Ocimum tenuiflorum* (L.)	ND	ND	ND
*Syzygium cumini* (L.) Skeels	ND	ND	44.37 ± 1.92*
*Azadirachta indica* Adr. Juss	41.7 ± 2.97**	62.43 ± 3.35*	ND
*Linum usitatissimum* (L.)	ND	ND	ND

The table shows percent inhibitory activity of small intestinal glucosidases using plant extracts. The data is indicated as the mean ± SEM; (*n* = 3). Two-tailed *t*-test was applied. Values with different asterisks (*,**) shows significant difference with (*) denoting more significant value, *P* <  .05, two-tailed student *t*-test. ND defines no enzyme inhibition.

**Table 3:** Percent enzyme inhibition shown by plant extracts with liver glucosidases.

Acarbose (95.61 ± 2.36)	(%) Glucosidases inhibition activity		
	Methanol	Aqueous	Chloroform
*Murraya koenigii* (L.) Sprengel	ND	ND	ND
*Bougainvillea spectabilis*	33.09 ± 3.02	ND	ND
*Ocimum tenuiflorum* (L.)	ND	26.65 ± 3.98	ND
*Syzygium cumini* (L.) Skeels	55.08 ± 3.32**	ND	ND
*Azadirachta indica* Adr. Juss	52.10 ± 3.31**	69.28 ± 2.59*	ND
*Linum usitatissimum* (L.)	ND	ND	ND

The table shows percent inhibitory activity of liver glucosidases using plant extracts. The data is indicated as the mean ± SEM; (*n* = 3). Two-tailed *t*-test was applied. Values with different asterisks (*, **) shows significant difference with (*) denoting more significant value, *P* <  .05, two-tailed student *t*-test. ND defines no enzyme inhibition.
